# “Smooth Muscle Cell Stiffness Syndrome”—Revisiting the Structural Basis of Arterial Stiffness

**DOI:** 10.3389/fphys.2015.00335

**Published:** 2015-11-18

**Authors:** Nancy L. Sehgel, Stephen F. Vatner, Gerald A. Meininger

**Affiliations:** ^1^Department of Cell Biology and Molecular Medicine, New Jersey Medical School, Rutgers University – Biomedical and Health SciencesNewark, NJ, USA; ^2^Department of Biomedical Engineering, New Jersey Institute of TechnologyNewark, NJ, USA; ^3^Dalton Cardiovascular Research Center, Department of Medical Pharmacology and Physiology, University of MissouriColumbia, MO, USA

**Keywords:** cell stiffness, atomic force microscopy, aorta, vascular smooth muscle cells, cell biology, cytoskeleton, collagen, elastin

## Abstract

In recent decades, the pervasiveness of increased arterial stiffness in patients with cardiovascular disease has become increasingly apparent. Though, this phenomenon has been well documented in humans and animal models of disease for well over a century, there has been surprisingly limited development in a deeper mechanistic understanding of arterial stiffness. Much of the historical literature has focused on changes in extracellular matrix proteins—collagen and elastin. However, extracellular matrix changes alone appear insufficient to consistently account for observed changes in vascular stiffness, which we observed in our studies of aortic stiffness in aging monkeys. This led us to examine novel mechanisms operating at the level of the vascular smooth muscle cell (VSMC)—that include increased cell stiffness and adhesion to extracellular matrix—which that may be interrelated with other mechanisms contributing to arterial stiffness. We introduce these observations as a new concept—the Smooth Muscle Cell Stiffness Syndrome (SMCSS)—within the field of arterial stiffness and posit that stiffening of vascular cells impairs vascular function and may contribute stiffening to the vasculature with aging and cardiovascular disease. Importantly, this review article revisits the structural basis of arterial stiffness in light of these novel findings. Such classification of SMCSS and its contextualization into our current understanding of vascular mechanics may be useful in the development of strategic therapeutics to directly target arterial stiffness.

## Introduction

Increased arterial stiffness is a significant consequence of the natural aging process, but it is also associated with the presence of underlying cardiovascular disease. As such, an increase in aortic stiffness is frequently observed in populations with hypertension, obesity and diabetes. Additionally, gender also plays a role in the manifestation of increased arterial stiffness, as women may be more vulnerable to its deleterious effects. Altogether, it is increasingly apparent that there is significant clinical value in assessing and fully understanding the phenomenon of increased vascular stiffness. A deeper understanding of the underlying mechanisms may help motivate future development of effective strategies to combat increased vascular stiffness by targeting novel cellular mechanisms and minimize associated cardiovascular disease risks.

While the topic of vascular stiffness has been previously reviewed, in this analysis, we focus on describing how cellular mechanics may contribute to the structural basis of increased arterial stiffness. In this review, we consider individual components of the vascular wall, and briefly present a literature survey to discuss current concepts of how these may contribute to vascular stiffness. We also emphasize in this review new reports of a stiff cellular phenotype that appears to impact endothelial cells (EC) and, as we have recently observed, vascular smooth muscle cells (VSMC). For VSMC, our work has demonstrated that there is a significant increase in the VSMC stiffness and adhesion to the extracellular matrix (ECM) - that occurs in conjunction with increased vascular stiffness. We hypothesize that this cellular level stiffening alters vascular function and contributes to the changes observed in vascular stiffening observed in aging and cardiovascular disease, which we term the “Smooth Muscle Cell Stiffness Syndrome.” Although, syndrome is technically defined as a group of symptoms that consistently occur together, we are using this term to describe the concurrence of vascular smooth muscle stiffness within the broader field of vascular stiffness. We believe that understanding the mechanisms that underlie this Syndrome will provide new clues to help improve our understanding and therapeutic targeting of increased vascular wall stiffness.

## Epidemiology of arterial stiffness

Changes in the mechanical properties of the vasculature are co-morbid with cardiovascular disease. Clinical studies have directly linked increased arterial stiffness to several risk factors for cardiovascular diseases—including hypertension (Avolio et al., 1985; Nichols et al., 1993; Tomiyama et al., 2004; Mitchell et al., 2010), aging (Newman and Lallemand, 1978; Franklin et al., 1997; Tomiyama et al., 2003; Matsuoka et al., 2005; Mitchell et al., 2007; AlGhatrif et al., 2013), atherosclerosis (van Popele et al., 2001; Gepner et al., 2014), obesity (Fu et al., 2013; Pal and Radavelli-Bagatini, 2013), diabetes (Shen et al., 2013; Shimizu et al., 2013), kidney disease (Baumann et al., 2014), and metabolic syndrome (Scuteri et al., 2014). In addition, there is evidence that increased aortic stiffness in woman is particularly predictive of eventual cardiovascular disease, and that women may be more vulnerable to the pathology associated with increased arterial stiffness (Scantlebury and Borlaug, 2011; Laurent et al., 2012a; Coutinho et al., 2013; Ben-Shlomo et al., 2014). There is a growing support for the concept that arterial stiffness is a good predicative biomarker for cardiovascular disease and risks (Vogel and Benitez, 2000; Anderson, 2006; Franklin, 2007; Laurent and Boutouyrie, 2007; Martin et al., 2008; Wang et al., 2008; Laurent et al., 2012a,b). Therefore, increased arterial stiffness should be considered as a biomarker central to aging and cardiovascular disease.

It is also well established that the presence of multiple cardiovascular risk factors may interact with and exacerbate increases in arterial stiffness. For example, there is considerable epidemiological evidence to suggest that hypertension accelerates the process of arterial stiffening in aging (Tomiyama et al., 2004; European Society of Cardiology, 2010; Verwoert et al., 2014). However, it has been controversial whether the risk factors directly cause the increase in arterial stiffness, or whether the increased vascular stiffness exacerbates the disease state, such as has been analyzed in the case of hypertension (Mitchell, 2014). Notably, a recent Framingham study found increased arterial stiffness precedes hypertension (Kaess et al., 2012), and this has supported the concept that arterial stiffness is not necessarily caused by hypertension but may be a good predictor of risk for hypertension development and its subsequent complications (Boutouyrie et al., 2002; Laurent et al., 2003; Tomiyama et al., 2013). It has also been conjectured that the presence of arterial stiffness may complicate the management of cardiovascular disease. For example, in patients with increased arterial stiffness, blood pressure is more difficult to control, as arterial stiffness is also associated with resistant hypertension (Pabuccu et al., 2012). However, the identity of the interactive mechanisms associated with arterial stiffness and cardiovascular risk factors remains unclear and may involve any number of environmental components within the vascular wall, such as mechanical stimuli, endothelial derived and inflammatory cytokines and changes in the properties of the ECM. Continued research is warranted to investigate the underlying mechanisms of arterial stiffness in cardiovascular disease, and how they interact with each other, as well as how systemic disease mechanisms influence the process of arterial stiffening.

## Pathophysiology of arterial stiffness

Vascular stiffness is considered a major health problem because it affects not only arterial pressure and is a cause of systolic hypertension and stroke, but also affects regulation of vascular resistance and blood flows to important regional beds including the coronary, renal and cerebral, which have obvious consequences when compromised. As discussed above, increased vascular stiffness is observed in many disease states, e.g., atherosclerosis, hypertension and diabetes, but it is important to note that it is most commonly found associated with aging. It is currently held that all elderly individuals suffer from increased vascular stiffness to a more or less degree. It is generally not recognized that vascular stiffness increases from fetal gestation to newborn to adults, even before the aging process begins (Pagani et al., 1979). The problem of increased vascular stiffness has been recognized for centuries, but techniques to assess its severity have changed. The most commonly used technique comes from autopsy and *in vitro* assessment of stiffness. *In vitro* measurements of stiffness are still widely employed. However, *in vivo*, techniques have also been applied. Most commonly finding increased systolic arterial pressure and a widened pulse pressure are used as indicators of increased aortic stiffness. More recently, with the advent of echo/ultrasound and MRI assessment of vascular dimensions, these techniques have been employed both in animal and human experiments to assess vascular stiffness. For many experiments in animals, anesthesia and recent surgery are required for these measurements, which by itself, has a major impact on vascular stiffness. Furthermore, the echo and MRI techniques have often only been used to obtain snapshots of vascular stiffness, not beat-to-beat changes. To obviate these problems, we developed techniques to measure vascular stiffness instantaneously and continuously in conscious animals without the complicating influences of recent surgery or anesthesia. To do this, ultrasonic crystals are implanted on opposing surfaces of the aorta and a pressure gauge is implanted in the aorta to provide beat by beat measurements of arterial pressure and diameter, which are critical for assessing stiffness (Pagani et al., 1978, 1979; Vatner et al., 1980). We have used these techniques for studies in monkeys, dogs, adult and newborn and fetal sheep (Pagani et al., 1978, 1979, 1980; Vatner et al., 1980, 1984; Macho and Vatner, 1981; Qiu et al., 2007, 2010) to overcome the limitations of other technical approaches.

It is clear that the pathophysiologic influence of increased arterial stiffness on the cardiovascular system is multifactorial. One major consequence of increase aortic stiffness is a diminished Windkessel effect within the cardiovascular system. First proposed by Frank in 1899, the Windkessel model reduces the vascular system into capacitance and resistance elements (Sagawa et al., 1990). In essence, the large arteries are functionally characterized as a capacitor or reservoir for ejected blood before it can by fully conducted through smaller resistance vessels. The aorta distends outwards to accommodate the surge in blood volume after it is ejected from the heart during systole, and subsequently recoils inward during diastole to continue to propel blood distally. This capacitive property of the aorta also extends the pressure gradient (as after the aortic valve closes, there is no upstream cardiac-facilitated pressure gradient) that aids in propelling the blood forward.

In addition, this capacitive behavior of the aortic wall also absorbs some of the energy during ejection and dampens the rise in systolic pressure thus modulating pulse pressure. As such, increased arterial stiffness also decreases the ability of the aorta to dampen the pressure pulse. The aorta normally acts to cushion the pressure pulse created by the ejected blood by absorbing energy. In doing so it acts to transform the ejected pulsatile flow into a more steady flow pattern as blood enters smaller vessels and downstream tissues. In the presence of increased stiffness of the aorta there is a diminished ability of the aorta to absorb energy following ejection and thus to diminish the amplitude of the pulse pressure and the transmitted arterial pressure wave. Consequently, an aorta with increased stiffness will exhibit a decrease in mechanical compliance, a diminished capacitance for ejected blood, less absorption of energy on ejection and result in a higher systolic pressure. This leads to an increased pulse pressure and deeper penetration of the pulse pressure wave into the peripheral vasculature. Frequently, increased arterial stiffness is associated with microvascular remodeling and impaired tissue blood flow autoregulation, eventually leading to end organ damage, which can be of particular clinical significance when the coronary, renal or cerebral circulations are impaired (Pessina, 2007; Safar and Lacolley, 2007; Mitchell, 2008; van den Akker et al., 2010). The role of the increase in pulse pressure in these microvascular changes and ultimate end organ damage requires further attention.

Finally, increased arterial stiffness is also damaging because it increases the workload on the heart. This increase in workload is derived from concepts concerning the importance of reflected waves within the arterial system (O'Rourke and Yaginuma, 1984; Latham et al., 1985; Nichols and Edwards, 2001). For every forward traveling pressure wave away from the heart, there a partially reflected pressure wave that travels backwards to the heart. A reflected wave is produced at sites of impedance discontinuity in the vascular system, which are typically occurring at branching points and high resistance/smaller caliber vessels (Nichols et al., 2005). Under normal conditions, the mechanical properties of the aorta are such that the return of the reflected wave occurs during diastole. This timing is physiologically beneficial to the heart since the coronary arteries are filled during diastole and as such the reflected wave contributes to enhanced filling of the coronary arteries. However, in an aorta characterized by increased stiffness, the reflected wave travels faster and reaches the heart during systole. This has the dual-fold effect of increasing afterload/workload and effectively and can lead to decreased stroke volume. Secondly, the mismatched timing of the return of the reflected wave with diastole decreases coronary artery perfusion. Numerous epidemiological studies have found an association between increased pulse wave reflection and cardiovascular disease (London et al., 2001; Nürnberger et al., 2002; Mitchell et al., 2004; van Trijp et al., 2004; Hashimoto et al., 2008; Nichols et al., 2013). Thus, the increase in aortic stiffness has an impact on both cardiac function and potentially important effects on the microcirculation and end organ functions.

## Arterial stiffness, a novel target for therapeutic control

Targeting arterial stiffness *per se* for therapeutic control represents a new avenue of cardiovascular disease. The concept of focusing directly on controlling vascular stiffness has its origins largely derived from studies by O'Rourke et al. on the control of hypertension (O'Rourke, 1985, 1989, 1990a,b, 1992; Avolio et al., 1986). The principle strategy has been utilization of existing antihypertensive therapies to control arterial stiffness (Safar et al., 1988). As previously reviewed by Benetos and colleagues, several classes of anti-hypertensive drugs—ACE inhibitors, calcium channel blockers, nitrates—have been demonstrated to have beneficial side-effects that include diminishing arterial stiffness (Benetos et al., 1997). However, these therapeutics were developed to decrease mean arterial pressure (MAP) by controlling total peripheral resistance (through acting as a VSMC relaxant), cardiac output, heart rate and blood volume, and thereby the effect on arterial stiffness is indirect and was not specifically targeted. The effectiveness of these drugs is, notably, varied, and still under evaluation (Briet and Schiffrin, 2013; Laurent and Boutouyrie, 2014; Peters et al., 2014).

Importantly, the selective use of anti-hypertensives to attempt to modulate arterial stiffness may not be adequate for all patients. For example, elderly patients often present with a decrease in diastolic pressure. This is an important therapeutic consideration, as the dosage for treatment with anti-hypertensives should only be administered to the point where diastolic pressure does not decrease below 70 mmHg (Kaplan, 2000). Thus, continuing to look at treatments that show increased selectivity for altering vascular stiffness would hold great potential. Moreover, the variability when comparing the effects of anti-hypertensive drugs on vascular stiffness suggests the presence of other unidentified mechanisms underlying arterial stiffness. Thus, a new generation of therapeutics that can selectively target arterial stiffness and control pulse pressure (PP), instead of the control of MAP may be of considerable value. Further, study in the identification of potential therapeutic targets to control arterial stiffness is warranted.

## Major mechanisms mediating causes of increased vascular stiffness

### Aging and gender considerations

**Figure 1:**
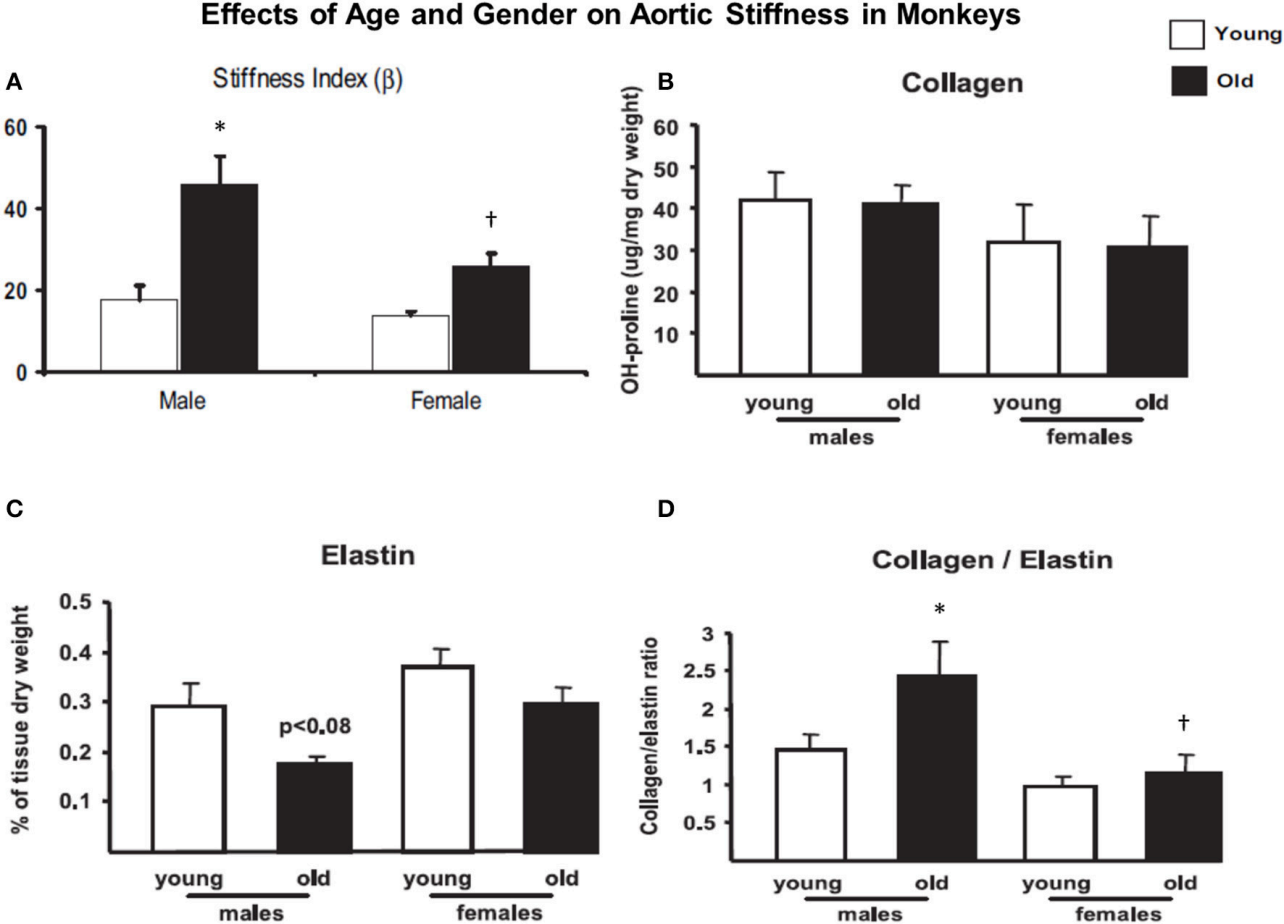
**(A)** Aortic stiffness increased in old males and females compared to younger monkeys. However, aortic stiffness was increased more in old males than old females. **(B)** There was little significant difference in collagen density in those groups. **(C)** Elastin density was decreased more in old males than the females. **(D)** The collagen/elastin ratio increased more in old males than old females. Reprinted from Qiu et al. (2007). ^*^P < 0.05 vs. corresponding young animals; ^†^*P* < 0.05 vs. corresponding old male monkeys.

As noted above the most common etiology of increased stiffness is aging. Most of the literature on this topic has used either human models of aging or animal models, most frequently rodents. Each of these have an inherent problem for aging studies. In aged humans, it is difficult to study the isolated effects of increased stiffness in the absence of some degree of atherosclerosis. We studied Fascicularis and Rhesus monkeys as young adults (3–7 years old) and after aging (over 20 years old). We found that aortic stiffness increased, but much more in old males than females (Figure 1), consistent with what is known in aging humans, where older females, before menopause, are protected from most cardiovascular diseases. Our data (Figure 1) and recent studies (Takagi et al., 2003; Qiu et al., 2007, 2010) demonstrate that there are major gender differences in aging monkeys, and that there are major differences in the expression of genes and proteins regulating vascular function that are evident even between young male and female monkeys (Qiu et al., 2007). Some prior studies have also found that older females are relatively protected from vascular stiffness (Jonason et al., 1997), and that this protection disappears after menopause (Waddell et al., 2001). Importantly, the physiology of female nonhuman primates is very similar to that of women in terms of reproductive adaptation to aging, including hormone profiles during the menopausal transition, extent of age-related and menopause-associated effects of changes in hormone levels on metabolism, bone loss, and impaired cardiovascular function (Rodgers et al., 1993; Walker et al., 1995; Gilardi et al., 1997; Qiu et al., 2007). The similarities also include the shifts in hormonal profiles through the menopausal transition, the progression to cycle termination through irregular cycles, and the protective responses to estrogen replacement following oophorectomy (Gilardi et al., 1997; Shideler et al., 2001; Cline and Wood, 2005). The use of rodents for these research purposes is limited by the fact that rodents do not have menstrual cycles and exhibit periods of persistent estrous, which is associated with elevated and constant levels of estrogen, low levels of progesterone, and a lack of luteinizing hormone surges (Felicio et al., 1984; Wu et al., 2005).

### Extracellular matrix

The most common mechanism thought to mediate increased vascular stiffness involves remodeling and content changes in the ECM of the aorta, with increases in collagen and decreases in elastin. Many studies can be found dealing with the structural content and properties of ECM proteins, predominantly collagen, elastin and fibronectin. Elastin is highly abundant in large and small arteries and in the aorta confers distensibility to account for the recoil ability of the aorta. At low mechanical loads, vascular mechanical properties are predominantly determined by elastin fibers. At these loads, collagen fibers have been histologically shown to have a loose, wavy configuration suggesting that they are not load-bearing. Upon higher mechanical loads, mechanical force transfers to the collagen fibers and they can be observed to straighten and lose their wavy appearance suggestive of bearing greater loads and thus contributing to a larger degree to the mechanical properties of the vessel. These relative mechanical contributions of elastin and collagen are thought responsible for the characteristic stress-strain curve of the arterial wall. Increased arterial stiffness has thus been proposed to be associated with increases in collagen content that occur concomitantly with decreases in elastin content. Numerous studies of aging vessels have reported increases in the collagen/elastin content ratio, and have associated this conclusion with the increase in arterial stiffness with aging. In fact, this view of increased collagen and decreased elastin has dominated thinking in the field of arterial stiffness to the point that it has become conceptually dogmatic.

However, an in depth analysis of published studies of hypertension strongly suggest that this view of an increased collagen to elastin ratio may not be entirely accurate. Several reports have demonstrated a lack of increased collagen in hypertension (Bezie et al., 1998a; Koffi et al., 1998, 1999; van Gorp et al., 2000; Sehgel et al., 2015), with some reporting decreases in vascular collagen (Cox, 1981; Mizutani et al., 1999). These stand in contrast to studies of hypertension that report observations of increased vascular collagen (Mourlon-Le Grand et al., 1993; Koffi et al., 1998, 1999), in a similar fashion as reported in studies of aging. A time-course study by Hu and colleagues found aortic collagen increased initially following induction of hypertension, but the concentration returned to levels not different from the sham control in the long-term (Hu et al., 2008). The inconsistency in the literature may reflect contributions from numerous variables between studies. For example, discrepancies in the quantification of arterial collagen, the models of hypertension used, the stage at which hypertension was studied and the precise vascular sites that were harvested for investigation (Sehgel et al., 2015). Importantly, studies of human essential hypertension, which assessed vascular collagen post-mortem, found that the collagen content was unchanged with hypertension (Schlatmann and Becker, 1977; Hoshino et al., 1995) or to have a similar concentration (Faber and Oller-Hou, 1952) compared to age-matched normotensive patients.

The rationale for examining mechanisms for increased vascular stiffness, apart from the extracellular matrix, at the level of VSMC was derived from our aging monkey studies where we observed no change in collagen density, but decreases in elastin were more marked in old males than old females (Figure 1). Below we review a number of the major causes that have been implicated as contributing to increased vascular stiffness. In reviewing these mechanisms we were surprised by the lack of consistent changes among various studies and that often there were no changes in collagen density. We hypothesized that the changes in elastin density were insufficient to cause such major changes in aortic stiffness. This led us to look for other causes of increased vascular stiffness resulting in our novel concept of increased stiffness at the level of individual vascular smooth muscle cells.

### Concept of increased vascular smooth muscle cells (VSMC) stiffness

**Figure 2:**
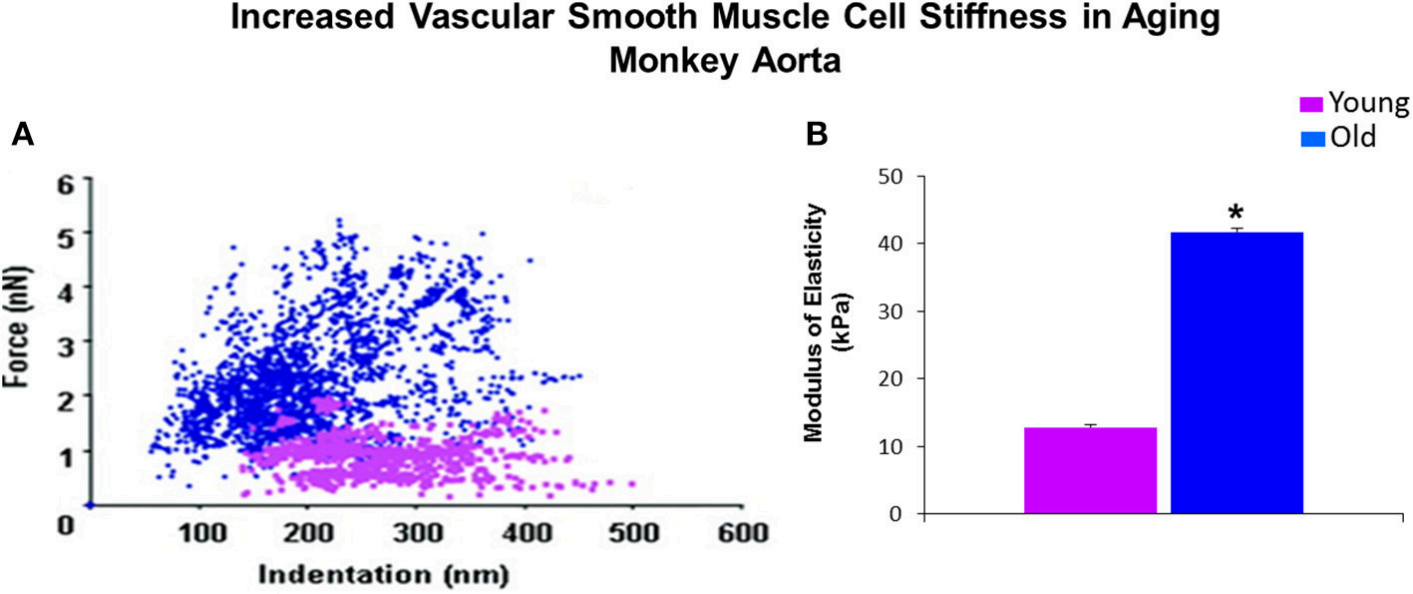
**Mechanical properties of single VSMC measured by AFM. (A)** Distribution of force as a function of indentation in young (pink) (*n* = 40 cells) and old (blue) (*n* = 76 cells) monkeys. Increased cell stiffness is evident as higher force requirement for indentation. **(B)** VSMC stiffness 4 fold increased in old vs. young monkeys. ^*^P < 0.05 vs. young monkeys. Reprinted from Qiu et al. (2010).

**Figure 3:**
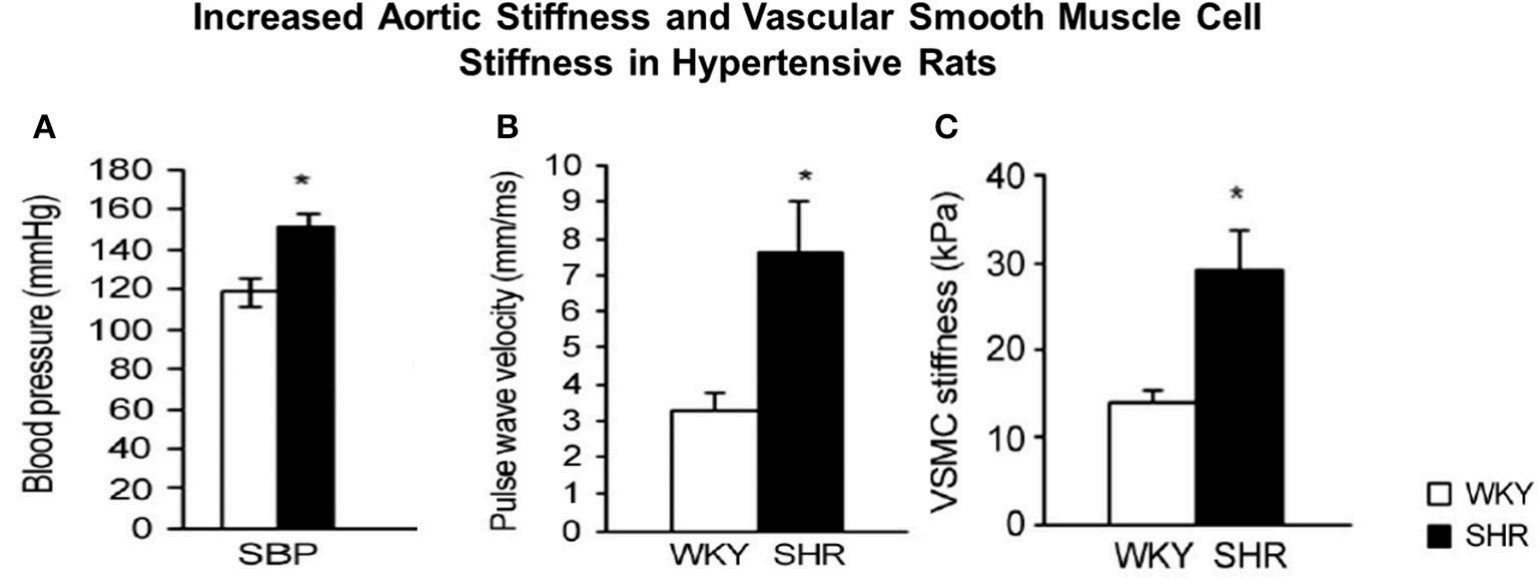
**Hypertension in spontaneously hypertensive rats (SHR) vs. Wistar-Kyoto (WKY) rats (A) was associated with increased aortic stiffness, measured by pulse wave velocity (B) and stiffness was also increased in individual vascular smooth muscle cells (VSMCs), as determined by atomic force microscopy measurements (C)**. ^*^P < 0.05, compared to WKY. Reprinted from Sehgel et al. (2013).

Although, as discussed, there are several components of the vessel wall that might induce and contribute to increased vascular stiffness. We have recently focused on the isolated VSMC from aging primates and used AFM to measure the stiffness of these isolated cells. Of relevance to this topic, we found that there was a major increase in VSMC stiffness (Figure 2). We then went on to study hypertension in SHR rats and confirmed that this mechanism is important for mediating increased stiffness, not only in aging, but also in hypertension (Figure 3).

Overall, there has been limited information available as to the contribution of VSMC to arterial stiffness and vessel wall mechanics. VSMC are the predominant cell type within the arterial wall, and in large arteries are estimated to account for 25–35% of total volume (Nichols et al., 2005). They play a critical role in regulation vascular tone that may impact importantly upon hypertension. In large arteries, VSMC are also responsible for buffering pressure pulsatility. Under chronic conditions, they respond to increased mechanical stress by undergoing hypertrophy (Li and Xu, 2000). Additionally, VSMC may also undergo a phenotypic switching from a quiescent contractile phenotype to a proliferative secretory phenotype. Recently, it has been suggested that VSMC may also dedifferentiate to a more osteogenic phenotype, and thereby may play a role in increasing stiffness through vascular calcification (Pikilidou et al., 2015). It is plausible that VSMC may play a larger role in contributing to vascular wall stiffness and arterial wall mechanics than previously considered.

However, a major limitation to investigations of VSMC contributions to arterial stiffness has been technical limitations associated with its independent measurement. There is the generally accepted assumption that the contribution of VSMC to vessel wall stiffness is related to the degree of active tone in the cell, which will stiffen the cell. However, an alternative possibility is that there may be more chronic changes in the inherent mechanical properties of VSMC themselves, independent of contractile tone, that lead to stiffening of the cell itself. For example, a change in the expression and elastic stiffness of the cellular cytoskeletal components that may in turn play a role in the stiffness of the vascular wall. There is abundant evidence to demonstrate that stiffness is an important functional property inherent to all cell types-both contractile and non-contractile (Schaer-Zammaretti and Ubbink, 2003; Defranchi et al., 2005; Lekka et al., 2005; Oberleithner, 2005; Trache et al., 2005; Cross et al., 2008; Wu et al., 2010; Barry et al., 2015).

**Figure 4:**
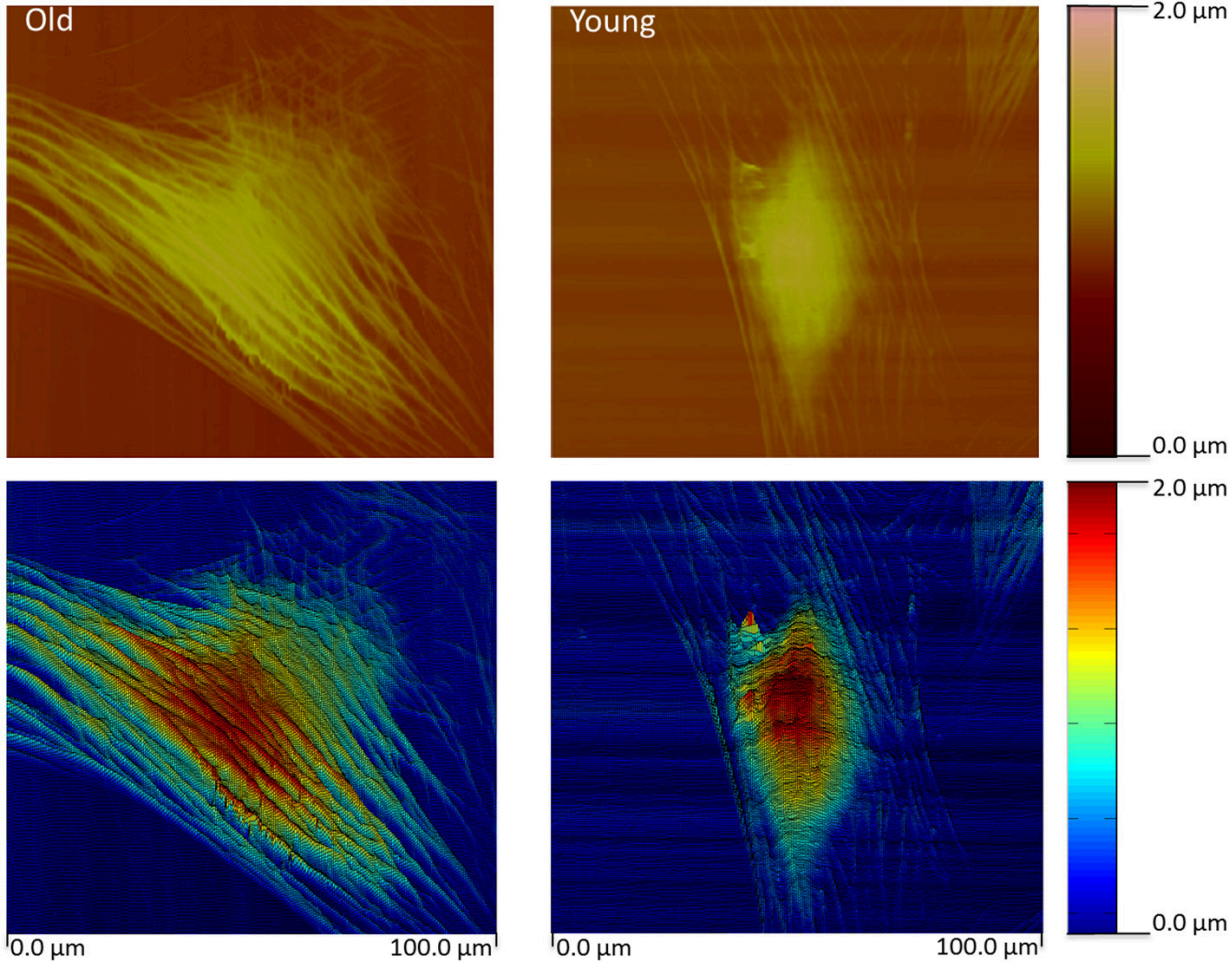
**Contact mode height image (top) for a typical old and young monkey aortic VSMC**. The height image data was pseudo- colored and slightly tilted to enhance relief contrast. The images reveal an extensive network of actin filaments in the old monkey VSMC compared to the Young monkey reflecting extensive cytoskeletal changes. Color bars index the cell height. Scale is given at the bottom. Reprinted from Zhu et al. (2012).

Using a specialized type of nanotechnology, AFM, we have recently reported the novel observation that intrinsic stiffness of VSMC is increased in association with increased aortic stiffness (Qiu et al., 2010; Zhu et al., 2012; Sehgel et al., 2013, 2015). This was accomplished using AFM probes to directly nanoindent the VSMC cell surface to permit estimation of the cell elastic modulus. The observed increase in VSMC stiffness was observed in resting cells independent of application of contractile agonists and was consistently found in several models exhibiting increased aortic stiffness: aging primates, hypertensive rats (spontaneously hypertensive rats (SHR)), and aging rats with and without hypertension. The increase in stiffness is accompanied by observable changes in the cytoskeletal features of the aged cells (Figure 4). We also noted the presence of increased stiffness in VSMC and overall vascular stiffness in aged rats without hypertension, supporting that increased VSMC stiffness reflects a feature of the aging vascular vasculature stiffness independent of arterial pressure (Sehgel et al., 2015). Collectively, the results of these studies strongly indicate that VSMC stiffness may be an important component of the increased vascular stiffness condition. Collectively, these observations point to the importance of developing a deeper understanding of factors and mechanisms that regulate cell stiffness in VSMC and how increased cellular stiffness affects VSMC function.

**Figure 5:**
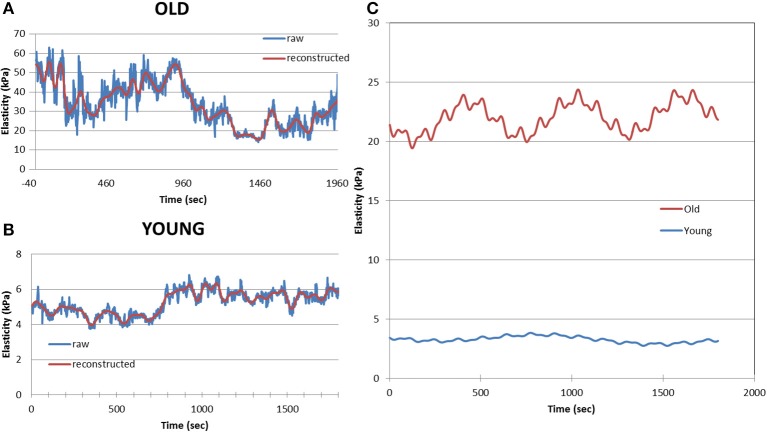
**Example of a 30 min records of cell elastic moduli for an old (A) and young (B) monkey VSMC**. Blue line shows raw data and the red line displays the reconstructed data after analysis. Data were processed using an eigendecomposition partition to permit the amplitude, frequency and phase components of the oscillation to be separated on cell-by-cell basis. Averaged data for old VSMC (*n* = 24) and young (*n* = 27) show differences in dynamic oscillatory behavior of old and young VSMC **(C)**. Reprinted from Zhu et al. (2012).

Of potential importance we observed that the stiffness of VSMC is a dynamic property, and it was observed to oscillate with time (Zhu et al., 2012; Sehgel et al., 2013). This dynamic behavior was altered in VSMC obtained from aged (Figure 5) and hypertensive animals (Zhu et al., 2012; Sehgel et al., 2013). This implies that dynamic mechanisms governing cytoskeletal properties, such as molecular motors and cytoskeletal polymerization/depolymerization, processes may be involved in increasing VSMC stiffness. However, additional work is required to fully understand these relationships. Altogether, increased stiffness at the level of the VSMC is a novel feature that appears to correlate with increased arterial stiffness. This represents a new potential mechanism that requires further consideration as a modulating factor contributing to arterial stiffness.

### Cell-matrix interactions

Cells are physically integrated within the vascular wall through adhesive interactions with tissue ECM involving integrins to form focal adhesions and with each other through cadherins that mediate cell-to-cell junctions. This part of the review will focus on the adhesion sites with the ECM that form part of an ECM-integrin-cytoskeletal axis that is responsive to the physical environment surrounding the cells. These interactions form the basis for a mechanical syncytium that contributes to regulation of the intrinsic tone and stiffness properties of the cell and vascular wall. VSMC are connected at multiple locations with the ECM. VSMC adhesion to fibronectin (FN) have been reported to be directly involved in mechanotranduction (Wang et al., 1993; Choquet et al., 1997; Riveline et al., 2001; Sun et al., 2008, 2012), and may underlie intrinsic myogenic tone in VSMC. It has also been demonstrated that when VSMC are exposed to vasoactive agonists that coordinated changes occur in the cell stiffness and the adhesion to ECM proteins (Hong et al., 2012, 2014, 2015). In addition, vasoactive agonists produce rapid remodeling of the cytoskeleton that is linked to changes in cell stiffness (Hong et al., 2014). This response may be related to the contractile function of VSMC and the proposal that transmission of generated force by VSMC to the ECM requires generalized strengthening of adhesions. However, it is also well documented that cells respond to stiffer ECM by becoming stiffer (Solon et al., 2007). Collectively, these data indicate that cells respond to both soluble factors and mechanical cues in their immediate environment and that impact VSMC mechanical and ECM adhesive properties. How these responses might also play a role in chronically elevating VSMC stiffness as is observed in cases of increased vascular stiffness remains to be determined, but the mechanisms that underlie these observations may provide fruitful areas for further investigation.

That adhesion to the ECM may be significant is supported by studies by Bezie et al. They have previously speculated that integrins may play an important role in vascular stiffness in hypertension. They observed an increase in the surface area on the VSMC membrane occupied by dense plaques and reported and increased expression of FN and α5 integrin in the aorta of hypertensive models with increased aortic vascular stiffness (Bezie et al., 1998a; Bézie et al., 1998b). Moreover, as we have previously noted (Sehgel et al., 2015), there are several reports of increased presence of FN and α5 integrin in the vessels of several hypertension models: SHR (Takasaki et al., 1990; Bezie et al., 1998a; Intengan et al., 1999), deoxycorticosterone/salt (Takasaki et al., 1990), angiotensin II-infusion (Takasaki et al., 1990), as well as non-hypertensive vascular stiffness models (Bouissou et al., 2014). Treatment with antihypertensive drugs has been reported to decrease vascular FN levels currently with a decrease in arterial stiffness (Koffi et al., 1998; Kakou et al., 2009). Morgan and colleagues have previously hypothesized that focal adhesions are a key regulator of vascular stiffness (Saphirstein et al., 2013; Gao et al., 2014; Saphirstein and Morgan, 2014), demonstrating an association between the modulation of cell contractility, cell stiffness, and focal adhesion size of VSMC. Collectively, these observations strongly suggest that alterations in the ECM adhesive properties of VSMC may also be an important site linked to changes in arterial stiffness.

We have recently reported increased adhesion to FN in VSMC derived from aging and hypertensive models with increased arterial stiffness (Qiu et al., 2010; Zhu et al., 2012; Sehgel et al., 2015). This was detected by using AFM probes coated with FN that were then brought into contact with the VSMC cell membrane surface. Analysis of the interactions between the AFM probe and the cell surface confirmed an increased adhesion of the FN coated AFM probe to VSMC derived from aging and hypertensive animals. We believe these measurements are among the first to directly confirm using biophysical assessment tools that increased attachment to FN does occur, and this property of VSMC is modified in the aging and hypertensive models. These observations further support the hypothesis that cell stiffness and adhesion are linked and may be important alternative therapeutic targets for to explore for anti-hypertensive therapy as well as anti-arterial stiffness therapy.

### Endothelial contributions

Endothelial mechanisms have also been proposed to contribute to arterial stiffness, namely through endothelial dysfunction leading to the decreased bioavailability of vasorelaxing factors such as nitric oxide (NO), endothelial derived hyperpolarizing factor and prostacyclin (Bellien et al., 2010; Sudano et al., 2011). Other endothelial factors such as endothelin-1 and C-type natriuretic peptide may also play a role (Vlachopoulos et al., 2010). Collectively, endothelial cells secrete vasoactive factors that function to regulate contractile tone in underlying VSMC. As reviewed by Wilkinson and colleagues, several conditions exhibiting endothelial dysfunction such as hypercholesterolemia and diabetes are also associated with increased arterial stiffness (Wilkinson et al., 2004). Arterial stiffness in patients has been shown to increase upon systemic administration of endothelial NO synthase inhibitors: L-NG-monomethyl arginine (LNMMA) (Wilkinson et al., 2002) and N(G)-nitro-L-arginine methyl ester (L-NAME) (McVeigh et al., 2001). As noted by Wilkinson and colleagues, though these infusions produce other changes in hemodynamics [e.g., increase in mean arterial pressure (MAP)] that confound the measurement of arterial stiffness, the contribution of NO has been validated by studies utilizing local administrations of NO synthase inhibitors (Wilkinson et al., 2004). Iliac artery stiffness was decreased and increased by local administration of glyceryl trinitrate and LNMMA, respectively, without any change in MAP (Schmitt et al., 2005). Similar studies demonstrated an opposite role for increased endothelin-1 in regulating vascular stiffness (McEniery et al., 2003).

One interesting hypothesis has been advanced by Oberleithner and colleagues has been that aberrant endothelial signaling may be the result of a mechanical stiffening of EC (Oberleithner, 2005; Fels et al., 2010, 2014; Kusche-Vihrog et al., 2015). The increased stiffness of the EC was associated with modulation of epithelial sodium channel (ENaC) activity and was associated with a reduced ability of the EC to produce NO (Kusche-Vihrog et al., 2010, 2014). This phenomenon has been termed the “stiff endothelial cell syndrome,” and has been speculated to characterize EC stress and thus contribute to vascular disease (Lang, 2011). It is possible that the increased stiffness of EC and subsequent reduction in production of vasorelaxing factors may also play a role in increasing vascular stiffness.

### Inflammatory mechanisms

Recently, the contributions of inflammatory mechanisms to arterial stiffness have attracted interest. Several clinical studies have established a link between increased arterial stiffness independent of other cardiovascular disease risk factors in patients with various inflammatory diseases, such as rheumatoid arthritis (Klocke et al., 2003) and inflammatory bowel disease (Korkmaz et al., 2014). Moreover, the higher arterial stiffness is ameliorated after treatments to control the inflammation, as shown by Zanoli and colleagues in patients with inflammatory bowel disease (Zanoli et al., 2014). As summarized by Jain and colleagues, in the short-term vascular stiffness may be increased due to the disruption of EC NO metabolism in inflammation, while in the long-term inflammation may lead to increased arterial stiffness through changes in collagen and elastin deposition and cross-linking (Jain et al., 2014). In experimental studies of hypertension, increased arterial stiffness has been shown to be mediated by T-cell mediated inflammation (Barhoumi et al., 2011; Wu et al., 2014). Altogether, inflammation also appears as a good candidate involved in altering vascular stiffness through fundamental mechanisms related to ECM modification, endothelial NO release and perhaps changes in VSMC function.

## Redefining the structural basis of arterial stiffness

As reviewed above, an increase in vascular stiffness can be observed in hypertension in in the absence of increased collagen content and decreased elastin content as the literature on this point is not consistent. This indicates that there are other operative mechanisms that require elucidation in the case of increased vascular stiffness. Furthermore, it also suggests the need to re-examine the temporal, structural and mechanical basis of arterial stiffness at smaller lengths of scales.

It is intriguing to speculate that perhaps structural explanations beyond changes in the ECM protein content and type for arterial stiffness have not been forthcoming for technical reasons. However, it is quite clear from the literature that the mechanical properties of the ECM must be considered. An appreciation for the pathophysiology of altered mechanical properties of arteries was first noted by Roy in the nineteenth century (Roy, 1881). After this, further recognition grew in the latter half of the twentieth century, and corresponded with the development of advanced measurement and analysis techniques (Nichols et al., 2005). Coincidently, during this period there were also a host of notable studies of arterial structure and geometry that provided detailed assessments and quantification of collagen and elastin within the arterial wall (Harkness et al., 1957; Wolinsky and Glagov, 1964; Fischer and Llaurado, 1966). In addition, it was determined that the elastic modulus of collagen is significantly greater than that of elastin (Dobrin and Canfield, 1984; Armentano et al., 1991). As recognized by Nichols and O'Rourke, when coupled with observations that the increase in the ratio of collagen-to-elastin down the arterial tree (Harkness et al., 1957) correlated with the increase in pulse wave velocity down the arterial tree (Nichols and McDonald, 1972), it became very plausible to suggest the hypothesis that an increase in collagen with a concurrent decrease in elastin was responsible for increased arterial stiffness.

**Figure 6:**
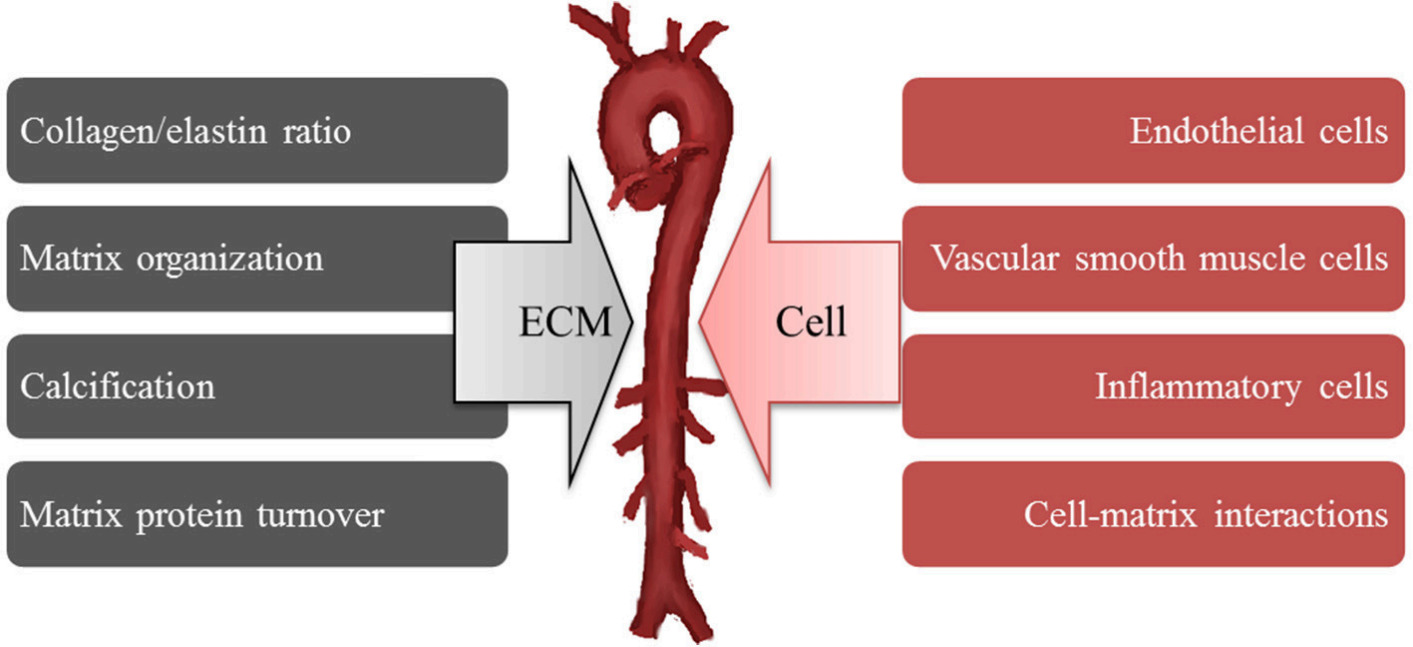
**Increases arterial stiffness develops from both extracellular (ECM) and cellular mechanisms**.

By extension, this structural mechanism has been applied to explain the increased arterial stiffness present in hypertension and this notion has persisted despite numerous controversial reports that do not necessarily support the proposed directionality of changes in collagen and elastin content in hypertension. Cox was one of the first to suggest that intrinsic differences in the mechanical properties of smooth muscle may be responsible for arterial stiffness. He based this conclusion on the finding of no innate difference in collagen and elastin coupled with the presence of increased stress development by vasoactive stimulation in hypertensive smooth muscle even after normalized to the smooth muscle content area (Cox, 1981). Despite knowledge that vasoregulatory agents can raise and lower vessel wall stiffness by modulation of VSMC tone, no consideration was given to idea that innate mechanical properties of VSMC (i.e., in an unstimulated or “passive” state) may be contributing to wall stiffness. In addition, little consideration was given to the possibility that adhesive coupling of the VSMC within the wall might be important. Such detailed study of cell mechanics have been hampered by the absence of adequate technologies for studies of the mechanical properties of single cells and molecules (e.g., AFM, magnetic bead twisting cytometry, micropipette aspiration, optical tweezers) that have only occurred in the last 50 years. The potential importance of mechanical contributions of VSMC to arterial stiffness has only recently been supported following the application of AFM to directly measure stiffness and adhesion in VSMC (Qiu et al., 2010; Zhu et al., 2012; Sehgel et al., 2013, 2015). However, this isolated cell data must ultimately be confirmed in the intact tissue and place into an integrated model of overall arterial wall tissue mechanics. It is clear that isolated cell mechanics are strongly influenced by the stiffness and type of underlying ECM substrate on which cells are grown. Also, isolating and placing cells in a culture environment can induce changes in cell phenotype. These facts underscore the need to continue to develop new model approaches to refine our understanding of how VSMC are affected by their surrounding ECM and mechanical environment and how VSMC contribute to the stiffness of the intact tissue. Continued development of appropriate methodologies, models and approaches will be required to fully address these issues. However, at this point, we would suggest that the determinants of increased arterial stiffness should be expanded to include the possible contributions of both extracellular and cellular mechanisms (Figure 6).

## Introducing the “smooth muscle cell stiffness syndrome” (SMCSS)

**Figure 7:**
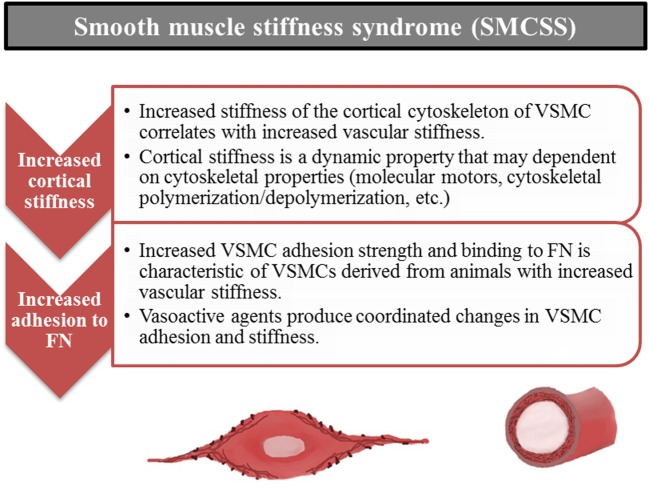
**Smooth muscle stiffness syndrome (SMCSS) is characteristic of increased arterial stiffness**. SMCSS describes the aberrant increased cortical stiffness and adhesion to fibronectin (FN) observed in vascular smooth muscle cells derived from stiff vessels. SMCSS may be an important therapeutic target for direct modulation of arterial stiffness.

The introduction of “Stiff Cell Syndrome” with respect to EC (Lang, 2011) and our recent findings for VSMC lead us to extend the concept of “Stiff Cell Syndrome” to include VSMC. This syndrome with respect to VSMC includes altered cellular mechanical and adhesive properties of the VSMC. It appears to involve cytoskeletal changes, focal adhesion changes and will perhaps be shown to involve cell-cell adhesion properties as well. Recognition that a smooth muscle cell stiffness syndrome may accompany increases in arterial stiffness opens up a variety of new investigative possibilities to improve our understanding of the mechanisms driving and underlying changes in arterial stiffness (Figure 7). We propose that stiffening of vascular cells importantly affects cell level functions, which in turn alters vascular functional and mechanical properties. As such, increased cell stiffness should be considered as part of the complex of changes observed in vascular stiffening that is observed in aging and cardiovascular disease.

## Conclusion

In conclusion we would suggest the presence of alternative mechanisms, rather than only changes in the ECM, as contributors to increased arterial stiffness. We introduce the concept of smooth muscle cell stiffness syndrome, to incorporate new information that increases in vascular stiffness are accompanied by increases in the stiffness and adhesiveness of VSMC at the cellular level. Importantly, modulation of these cellular mechanisms may be an opportunistic target toward the development of novel pharmacological therapeutics for hypertension and arterial stiffness.

### Conflict of interest statement

The authors declare that the research was conducted in the absence of any commercial or financial relationships that could be construed as a potential conflict of interest.
